# Diagnostic Accuracy and Incremental Value of Contrast-Enhanced Mammography Compared With Full Field Digital Mammography in a Tertiary Cancer Care Center

**DOI:** 10.7759/cureus.68601

**Published:** 2024-09-04

**Authors:** Palak Popat, Venugopal Prudveesh Kumar Reddy Nandi, Aparna Katdare, Purvi Haria, Meenakshi Thakur, Suyash Kulkarni

**Affiliations:** 1 Department of Radiodiagnosis, Tata Memorial Hospital, Tata Memorial Centre, Mumbai, IND

**Keywords:** contrast-enhanced digital mammography, contrast-enhanced mammography, contrast-enhanced spectral mammography, sono-mammography, mammography, cesm, cedm, cem

## Abstract

Objective: To assess the diagnostic accuracy and incremental value of contrast-enhanced mammography (CEM) compared with full-field digital mammography (FFDM).

Methodology: A retrospective analysis was performed with 150 consecutive patients who underwent CEM at our institute between November 2020 and February 2021, fulfilling the inclusion criteria. The first round of analysis included a review of FFDM with an interpretation of findings as per the Breast Imaging Reporting and Data System (BIRADS) lexicon and the assignment of the BIRADS category to the detected abnormalities. After this documentation, a second round of analysis included a review of recombined subtracted images of CEM. The diagnostic accuracy of FFDM and CEM was calculated with histopathology as the gold standard.

Results: Among the 150 cases assessed, 202 lesions were detected with histopathological correlation, of which 42 were benign and 160 were malignant. The sensitivity of FFDM was 90.6% compared to 98.12% for CEM. The specificity of FFDM was 66.7% compared to 76.19% for CEM. The negative predictive value (NPV) of FFDM was low, at 65.12%; CEM showed a better NPV, at 91.43%. The positive predictive value (PPV) was almost the same, at 94.01% for CEM and 91.19% for FFDM. The area under the curve (AUC) was superior for CEM compared to that of FFDM, with a value of 0.87. FFDM had a low sensitivity, especially in dense breast parenchyma, at 88.79% and a specificity of 70%, whereas CEM showed a higher sensitivity, specificity, and NPV, measuring 99.14%, 76.67%, and 95.83%, respectively.

Conclusion: Superior sensitivity and high NPV for CEM make it a preferable modality compared with FFDM, especially in dense breast parenchyma, where CEM overcomes the limitations of FFDM. We conclude that CEM is superior to FFDM in evaluating the extent of disease, additional satellite lesion detection, and ruling out ambiguous findings.

## Introduction

Breast cancer has pivoted at the center of a triangle of screening, diagnosis, and management. Currently, full-field digital mammography (FFDM) remains the preferred first diagnostic modality in suspected breast cancer [1] and is integrated into national health programs for breast cancer screening in many developed nations. Early breast cancer detection in routine screening FFDM has been a factor in decreasing mortality by almost 40% in women aged 40-74 years [2,3]. The overall sensitivity of FFDM is 75% and the specificity is 71% [4,5]. Despite its widely acknowledged value in detecting and characterizing breast pathologies, FFDM has its own limitations, with a false-negative rate ranging from 4% to 34% and decreased sensitivity in women with radiographically dense breast parenchyma [6-8].

In view of a lack of implementation of national-level mammography screening programs, the utility of FFDM in low-middle-income countries has been reserved for diagnosis. In this scenario, FFDM is usually performed on symptomatic and high-risk women, and this subset requires additional imaging such as ultrasound sonography (USG) or magnetic resonance imaging (MRI) for a complete assessment.

Early detection, diagnosis, and accurate assessment of disease extent and appropriate management are essential to achieve disease-free intervals and cancer-specific survival. Breast conservation surgery (BCS) offers superior quality of life and lesser morbidity of nodal dissection. The success of BCS in avoiding residual and recurrent disease rests with the radiologist in determining the true extent of the disease by detecting impalpable suspicious additional lesions, which can alter the area of surgical resection. In dense breasts, the latter are often missed in FFDMs. For this reason, supplemental imaging with USG has been widely accepted and performed; however, both FFDM and USG have limitations and require additional physiological/functional imaging such as dynamic contrast-enhanced MRI.

The limitations of MRI are high false positive rates, higher costs and lesser availability, and a need for a greater learning curve for interpretation [6,9]. These limitations explain the restriction of access to additional imaging and portend a need for more economical, easier-to-interpret, and more readily available investigation.

One novel imaging technique that fits those requirements is contrast-enhanced mammography (CEM). CEM is an emerging technique upgrade to FFDM, which improves FFDM’s anatomical imaging ability to provide functional imaging through the administration of intravenous contrast agents to identify abnormalities based on angiogenesis [7]. CEM appears to have a good prospectus to bridge the gap between FFDM and MRI, with a shorter examination time than MRI, acquisition simultaneous with FFDM, and a far lower cost than MRI [10]. CEM is a new modality not yet integrated into standard breast cancer management and lacks established indications.

We conducted a retrospective study to analyze the diagnostic accuracy and incremental value of CEM as compared with FFDM in a tertiary cancer care center.

## Materials and methods

A retrospective study was conducted at Tata Memorial Hospital, a tertiary cancer care hospital in Mumbai, India, following approval from the Institutional Review Board, which constitutes the Institutional Ethics Committee - Tata Memorial Centre (approval no. 3712). The study was conducted in accordance with the 2006 guidelines of the Indian Council of Medical Research.

Eligibility criteria for the study included patients who had undergone CEM in our institute as a part of their diagnostic workup from November 2020 to February 2021. The patients’ FFDM and CEM images were available in the picture archiving and communicating system (PACS) and their histopathological findings were documented in electronic medical records (EMR). CEM is performed as a supplemental modality when indicated, such as in heterogenous and dense breasts that have inconclusive findings in FFDM, persistent asymmetries, and high-risk cases, with the purpose of detecting primary and additional FFDM-occult lesions and determining the extent of disease. About 159 patients underwent CEM at our institute during the mentioned time period. Nine of these patients had no histopathological findings documented on EMR and were excluded from the study. The retrospective study was performed with 150 patients fulfilling the inclusion criteria.

**Figure 1:**
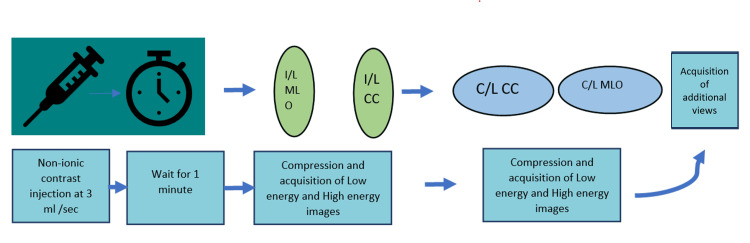
Flow chart representing the sequence of acquisition of CEM images I/L: ipsilateral; C/L: contralateral; CC: craniocaudal view; MLO: mediolateral oblique view

As per our institute’s protocol, normal serum creatinine and informed consent were taken. Additionally, a history of contrast allergy was ruled out. A non-ionic low-osmolar iodinated contrast agent was administered intravenously at a dose of 1.5 mL/kg and a rate of 3 mL/sec. Low- and high-energy images were acquired in bilateral craniocaudal (CC) and mediolateral oblique (MLO) views between two to eight minutes after contrast agent administration [11]. Low-energy images (26-32 kVp) were acquired at kilovoltage below the K-edge of iodine. Iodine was not attenuated in low-energy images; hence, the low-energy images were nearly identical to standard digital mammograms. High-energy images (45-49 kVp) were acquired above the K-edge of iodine. Iodine and breast parenchyma, both attenuated in the high-energy images, were thereby rendered noninterpretable. The low- and high-energy images were post-processed to obtain recombined subtracted images, depicting areas of contrast enhancement, by suppressing the density from the background breast parenchyma. Figure 1 depicts a flow chart representing the sequence of acquisition of CEM images.

The study was performed by evaluation of Digital Imaging and Communications in Medicine (DICOM) images of FFDM and CEM available in PACS by an expert in breast imaging, who had experience of more than 10 years. All cases were analyzed with the reader blind to previous diagnostic reports available on EMR. The first round of analysis included a review of FFDM with the interpretation of findings as per the Breast Imaging Reporting and Data System (BIRADS) lexicon and assigning of BIRADS category to the detected abnormalities. After this documentation, a second round of analysis included a review of recombined subtracted images of CEM. Due to the paucity of the available standardized lexicon at the time of the study, the interpretation and nomenclature of the CEM findings were interpolated from the MRI-BIRADS lexicon and assigned a BIRADS category accordingly. There is no washout period between the two rounds of analysis. If additional USG images were available, their implication in locoregional assessment and management was assessed and documented. The gold standard was the histopathology report obtained either after surgery, biopsy, or fine needle aspiration cytology (FNAC). diagnostic accuracy for the abnormalities detected on imaging was compared with the histopathology, with dichotomization of the BIRADS category such that BIRADS 4 and 5 lesions were considered malignant, while BIRADS 3 or less were classified as benign.

The extent of disease was available in the histopathology of 66 patients who underwent surgical resection. In this subset, the mean and extent of disease were calculated via various modalities of imaging and histopathology.

The recently published CEM-BIRADS lexicon [12] was not available at the time of our study; therefore, the study was predominantly based on interpolated MRI-BIRADS lexicon. As per the lexicon, low-energy images of CEM remained the same as mammography, while terms were adopted from

MRI and modified to cover situations unique to CEM in the case of recombined subtracted images. Additionally, we applied a similar strategy to our nomenclature, with a 90% congruence between our methodology and the CEM-BIRADS lexicon in terms of nomenclature and interpretation. The similarity was in the following nomenclature: breast composition, background parenchymal enhancement, mass (shape, margin, and internal enhancement characteristics), and non-mass enhancement (NME). The term grading and extent of enhancement were the only nomenclatures that were not part of our study; however, they were part of our interpretation where non-circumscribed enhancement extending beyond the mammographic visible lesion was designated as NME and included in disease extent.

Statistical analysis

Data were descriptively analyzed using mean and standard deviation, with median and interquartile range calculated for continuous variables and frequency and proportion computed for categorical variables. The overall diagnostic accuracy of CEM in detecting malignant lesions was compared with FFDM and USG using measures such as sensitivity, specificity, negative predictive value (NPV), positive predictive value (PPV), and receiver operating characteristic curve (ROC). Using Pearson’s correlation coefficient, the extent of disease in each modality was compared with the extent of disease available on the histopathology reports after surgical resection in a subset of cases.

## Results

To assess the diagnostic accuracy and incremental value of CEM as compared with FFDM, a retrospective analysis was performed on patients having undergone CEM. The mean age of these patients was 48 (range: 25-71) with approximately 100 patients in the 41-60 age range. Analyzing the eligible 150 patients, 84 had multiple findings, each requiring dedicated characterization. Hence, each lesion was conferred a unique existence, resulting in a total of 250 lesions. Of these 250 lesions, 202 had histopathological correlation and were included in the study. About 147 of the 150 patients underwent additional USG, demonstrating 198 lesions with histopathological correlation.

The lesions with no histopathological correlation were satellite lesions <5 mm from the index lesion, either not mentioned separately on the histopathology reports (9/48), lesions not seen post-chemotherapy (31/48), or benign and followed up (8/48).

The distribution of parenchyma was predominantly type C or type D, as seen in 105 of the 150 cases (70%).

Distribution of type A, B, C, and D parenchyma is 1 (0.7%), 44 (29.3%), 74 (49.3%), and 31 (20.7%), respectively.

**Figure 2:**
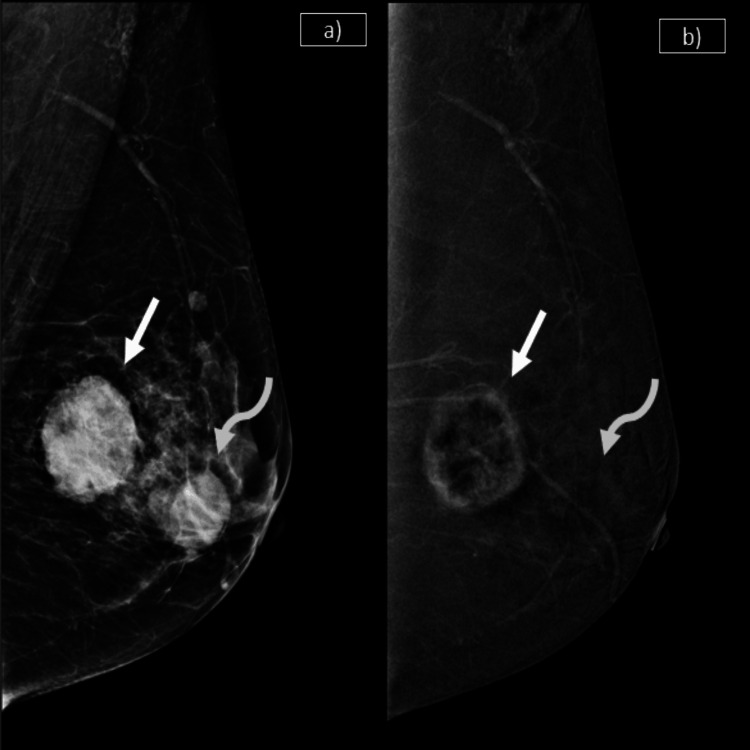
Co-existing malignant and benign pathologies, and their differentiation in FFDM and CEM a) Low-energy image on the left shows irregular mass with micro-lobulated margins seen posteriorly (solid arrow). Another oval mass with obscured margins is seen anteriorly in the central region (curved arrow). b) The recombined subtracted CEM images on the right show peripheral rind of heterogeneous enhancement in the posterior-most lesion (solid arrow). The other lesion shows no significant enhancement (curved arrow). The enhancing lesion corresponds to invasive breast carcinoma and the non-enhancing lesion corresponds to fibroadenoma. CEM: contrast-enhanced mammography; FFDM: full-field digital mammography

Of the 202 lesions, most were shown to be malignant in the histopathology (n = 160) and the rest were benign (n = 42). Table 1 shows the distribution of various histological types of malignant and benign lesions. Based on the distribution of histological type in the pathology, invasive breast carcinoma of no special type (IBC-NST) was the most common malignant pathology, seen in 139 lesions, and fibrocystic changes were the most common benign pathology, seen in 10 lesions. Figure 2 shows co-existing malignant and benign pathologies and their differentiation in FFDM and CEM.

**Table 1:** Distribution of various histological types of benign and malignant lesions DCIS: ductal carcinoma in situ; LCIS: lobular carcinoma in situ

Benign pathologies		Malignant pathologies	
Benign breast tissue	4	DCIS	5
Benign phyllodes tumor	2	LCIS	1
Fibroadenoma	9	Invasive lobular carcinoma	5
Fibrocystic change	10	Invasive breast carcinoma	139
Flat epithelial atypia	1	Invasive micropapillary carcinoma	1
Hamartomatous Fibroadenoma	2	Metaplastic carcinoma	1
Inflammation	1	Metastatic adenocarcinoma from ovary	3
Necrosis	1	Microinvasive breast carcinoma	1
Necrotizing granulomatous inflammation	2	Mucinous carcinoma of breast	1
No residual tumor in cases post-excision biopsy	3	Mucinous micropapillary carcinoma	1
Papilloma	1	Solid papillary carcinoma	1
Sclerosing adenosis	5	Undifferentiated round cell carcinoma	1
Hemorrhagic cystic cavity	1		
Total	42	Total	160

Diagnostic accuracy of CEM, FFDM, and USG

**Figure 3:**
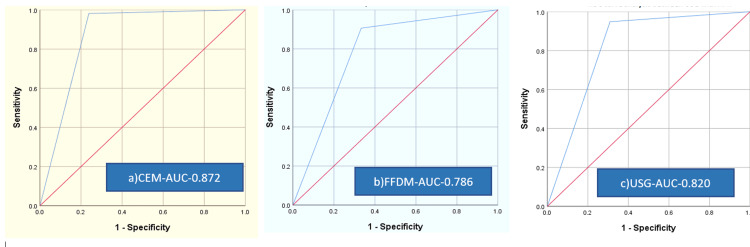
ROC curve analysis Graph showing ROC curve analysis between CEM, FFDM, and USG with histopathology. a) AUC of CEM - 0.872; b) AUC of FFDM - 0.786; c) AUC of USG - 0.820. P-value < 0.001. AUC: area under the curve; ROC: receiver operating characteristic curve; CEM: contrast-enhanced mammography; FFDM: full-field digital mammography; USG: ultrasound sonography

CEM had superior sensitivity, specificity, PPV, and NPV in comparison with FFDM, with values of 98.12%, 76.19%, 94.01%, and 91.43%, respectively. The overall diagnostic accuracy of CEM was 93.56%. The sensitivity and specificity of CEM were greater than FFDM, with a difference of 8% and 10%, respectively. The NPV of CEM was far superior to FFDM, with a difference of 26.31%, signifying CEM’s additive value. Table 2 shows the correlation between the BIRADS category and histopathology. Additionally, the area under the curve (AUC) of CEM was greater than FFDM and classified with good accuracy with a measurement of 0.872 (p-value < 0.001). Figure 3 shows the ROC curve analysis between CEM, FFDM, and USG with histopathology. 

**Table 2:** BIRADS category and correlation with histopathology FFDM: full-field digital mammography; CEM: contrast-enhanced mammography; BIRADS: Breast Imaging Reporting and Data System

	FFDM			CEM		
BIRADS	Benign	Malignant	Total	Benign	Malignant	Total
0	12	14	26	3	1	4 (1.9%)
1	1	0	1	1	0	1 (0.5%)
2	5	0	5	19	0	19 (9.4%)
3	9	0	9	8	2	10 (4.9%)
4A	8	5	13	5	1	6 (2.9%)
4B	1	6	7	0	1	1 (0.5%)
4C	6	69	75	4	58	62 (30.6%)
5	0	66	66	2	97	99 (49%)
	42	160	202	42	160	202

Seventeen lesions with histopathology not identified in the FFDM were seen in the CEM. Twelve were malignant and the remaining five were benign. In the CEM, nine of the 17 were seen as masses, seven as NME, and one as solitary enhancing focus.

In a subset analysis of patients with USG findings available, 147 patients with 198 lesions had histopathological correlation. USG had a superior sensitivity compared to FFDM; however, CEM surpassed USG with a sensitivity of 98.12% compared to 94.87%. The specificities of CEM and USG were 69.05% and 76.19%, respectively. Like FFDM, CEM had a far superior NPV at 91.43% in comparison with USG at 78.38%. Further, the PPV of CEM was superior to that of USG, with a positive difference of 4%.

Tables 3-7 show the results of the study, BIRADS category assigned in each modality, diagnostic accuracy of individual modality, and intermodality comparison.

**Table 3:** Cross-tabulation of CEM and histopathology findings for estimation of diagnostic accuracy CEM: contrast-enhanced mammography; HPR: histopathology; M: malignant; B: benign

	HPR		
CEM	M	B	Total
Malignant	157 (77.7%)	10 (4.9%)	167
Benign	3 (1.5%)	32 (15.8%)	35
Total	160	42	202

**Table 4:** Cross-tabulation of FFDM and histopathology findings for estimation of diagnostic accuracy FFDM: full-field digital mammography; M: malignant; B: benign; HPR: histopathology

	HPR		
FFDM	M	B	Total
M	145 (71.7%)	14 (6.9%)	159
B	15 (7.4%)	28 (13.8%)	43
Total	162	42	202

**Table 5:** Crosstabulation of USG and histopathology findings for estimation of diagnostic accuracy USG: ultrasound sonography; M: malignant; B: benign; HPR: histopathology

	HPR		
USG	M	B	Total
M	148 (74.7%)	13 (6.5%)	161
B	8 (4.0%)	29 (14.6%)	37
Total	156	42	198

**Table 6:** Comparison of diagnostic accuracy of CEM, FFDM, and USG CEM: contrast-enhanced mammography; FFDM: full-field digital mammography; USG: ultrasound sonography; PPV: positive predictive value; NPV: negative predictive value

	CEM		FFDM		USG	
Statistical parameters	Value	95% CI	Value	95% CI	Value	95% CI
Sensitivity	98.12%	94.62% to 99.61%	90.62%	85.01% to 94.66%	94.87%	90.15% to 97.76%
Specificity	76.19%	60.55% to 87.95%	66.67%	50.45% to 80.43%	69.05%	52.91% to 82.38%
PPV	94.01%	90.13% to 96.43%	91.19%	87.07% to 94.09%	91.93%	87.86% to 94.71%
NPV	91.43%	77.44% to 97.07%	65.12%	52.42% to 75.97%	78.38%	64.18% to 88.00%
Accuracy	93.56%	89.25% to 96.53%	85.64%	80.04% to 90.17%	89.39%	84.25% to 93.31%

**Table 7:** Comparison of AUC of FFDM, CEM, and USG ^a^: under the nonparametric assumption; ^b^: null hypothesis: true area = 0.5 CEM: contrast-enhanced mammography; FFDM: full-field digital mammography; USG: ultrasound sonography

	Area under the curve (AUC)				
	Area	Std. error^a^	Asymptotic sig.^b ^ (p-value)	Asymptotic 95% confidence interval	
				Lower bound	Upper bound
FFDM	0.786	0.046	<0.001	0.697	0.876
CEM	0.872	0.040	<0.001	0.793	0.950
USG	0.820	0.044	<0.001	0.732	0.907

**Figure 4:**
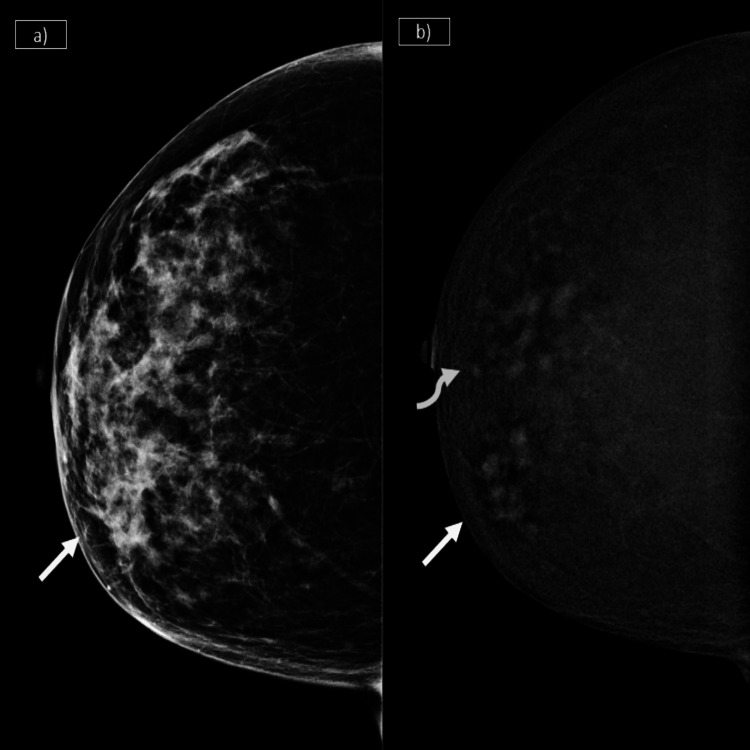
a) Irregular high-density mass (solid arrow) with indistinct margins is seen in the lower inner quadrant and central region in FFDM. b) In the subtracted CEM images, extent and additional satellite lesions (curved arrow) are seen clearly, in this case with dense breast parenchyma CEM: contrast-enhanced mammography; FFDM: full-field digital mammography

FFDM had a low sensitivity, especially in dense breast parenchyma, at 88.79% and a specificity of 70%, whereas CEM showed a higher sensitivity, specificity, and NPV, measuring 99.14%, 76.67%, and 95.83%, respectively. This shows a remarkably better performance in dense breast parenchyma on the part of CEM. Figure 4 represents the ability of CEM to detect additional satellite lesions occult in FFDM in dense breasts. In contrast, in patients with scattered fibroglandular parenchyma, the sensitivity of CEM was similar to that of FFDM and USG; however, CEM had a superior specificity (75%; +17% over FFDM) due to its ability to characterize lesions as benign or malignant based on enhancement characteristics. Table 8 highlights the comparison of diagnostic accuracy of CEM, FFDM, and USG in type A and B parenchyma and type C and D parenchyma.

**Table 8:** Comparison of diagnostic accuracy of CEM, FFDM, and USG in type A and B parenchyma and type C and D parenchyma ^*^ indicates that the values are dependent on disease prevalence CEM: contrast-enhanced mammography; FFDM: full-field digital mammography; USG: ultrasound sonography; PPV: positive predictive value; NPV: negative predictive value

	Scattered fibroglandular parenchyma (type A and B)			Dense breast parenchyma (type Cand D)		
Statistical parameters	FFDM	CEM	USG	FFDM	CEM	USG
Sensitivity	95.45%	95.45%	95.24%	88.79%	99.14%	94.74%
Specificity	58.33%	75.00%	75.00%	70.00%	76.67%	66.67%
PPV (^*^)	89.36%	93.33%	93.02%	91.96%	94.26%	91.53%
NPV (^*^)	77.78%	81.82%	81.82%	61.76%	95.83%	76.92%
Accuracy (^*^)	87.50%	91.07%	90.74%	84.93%	94.52%	88.89%

**Figure 5:**
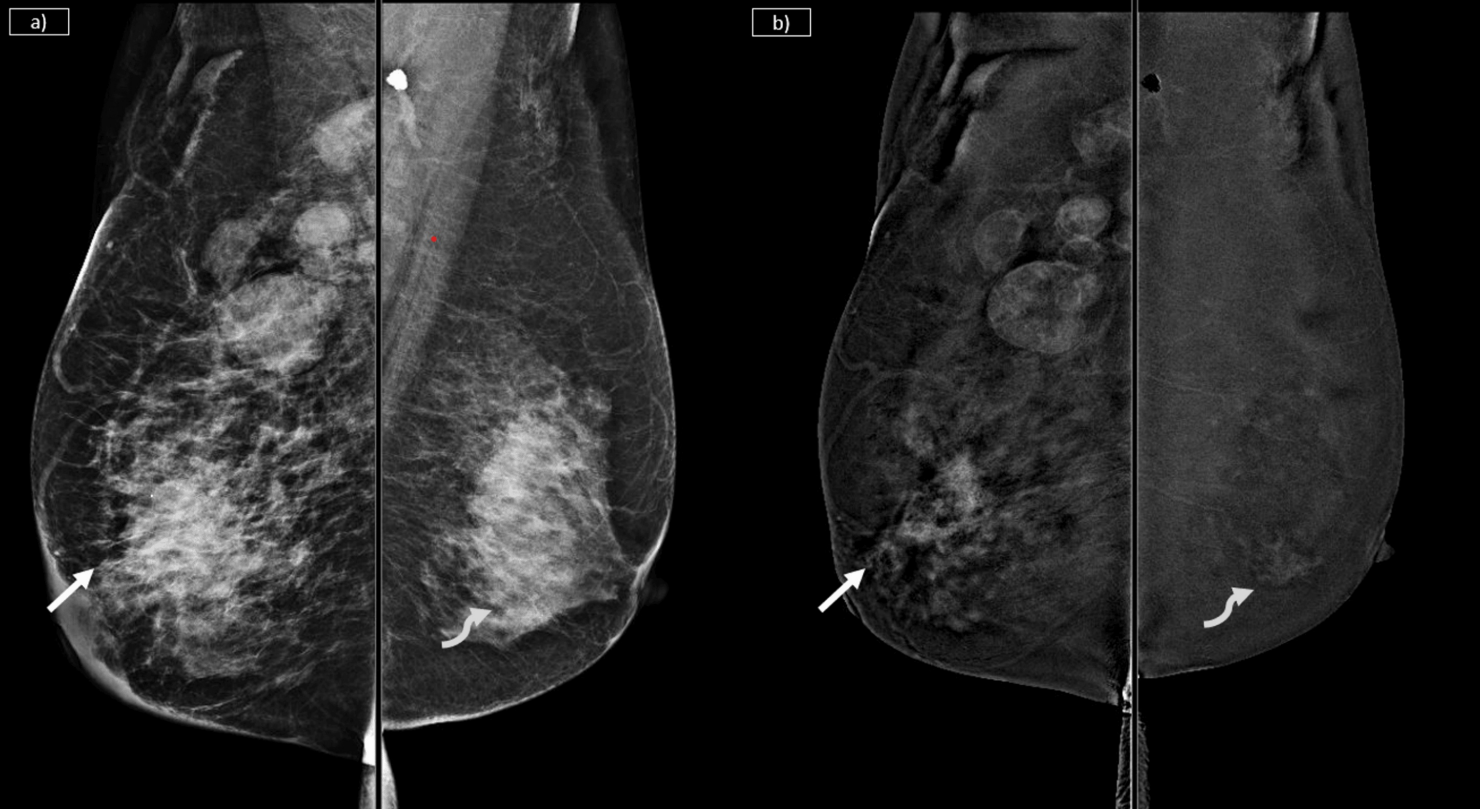
a) Ill-defined high-density mass (solid arrow) with indistinct margins seen in upper aspect and central region, with skin thickening and enlarged axillary nodes in the right breast in FFDM. Monomorphic calcifications are seen in the lower aspect of the left breast on MLO view in FFDM (curved arrow). b) Ill-defined lesion on subtracted images of CEM shows heterogenous enhancement in the upper central region (solid arrow) with metastatic axillary nodes. The area corresponding to monomorphic calcifications in the left breast shows NME (curved arrow) and was confirmed as microinvasive breast carcinoma on histopathology FFDM: full-field digital mammography; CEM: contrast-enhanced mammography; MLO: mediolateral oblique

The benefits of CEM as compared with FFDM were analyzed for individual patients and not lesions, where the interpretation would have a role in further management. In comparison with FFDM alone, various incremental benefits were observed in 121 of the 150 patients. In the remaining 29 patients, CEM was concordant with FFDM with no additional benefit. This benefit was predominantly observed in the superior assessment of disease extent in 74 patients, additional lesion detection in 54 patients, confirmation of spurious asymmetric densities in FFDM as usual parenchyma in 100 patients, correct characterization of a lesion as benign or malignant in 46 patients, and estimation of residual viable disease in post-neoadjuvant chemotherapy in 13 patients. Figures 5a-5b show the benefit of CEM in evaluating disease extent in the ipsilateral breast and identifying occult malignancy on the contralateral side.

Correlation of extent of disease

The correlation of extent of disease was superior in CEM compared with other modalities, where the correlation coefficient for CEM-determined extent of disease versus histopathology had a value of 0.707 (p < 0.001), and the other modalities, FFDM and USG, recorded 0.464 (p < 0.001) and 0.480 (p < 0.001), respectively. In patients with microcalcifications, the estimation of the extent of disease was superior with a combination of the extent of enhancement in CEM and the extent of microcalcifications in FFDM. The correlation coefficient of the extent of disease in various modalities of imaging and histopathology was calculated as shown in Table 9.

**Table 9:** Comparison of correlation of extent of disease in CEM, FFDM, and USG with histopathology CEM: contrast-enhanced mammography; FFDM: full-field digital mammography; USG: ultrasound sonography

	Comparison	Number of cases	Correlation coefficient	p-value
1	CEM extent of disease and HPR extent of disease	66	0.707	<0.001
2	FFDM extent of disease and HPR extent of disease	66	0.464	<0.001
3	USG extent of disease and HPR extent of disease	63	0.480	<0.001

## Discussion

In 2011, the United States Food and Drug Administration (FDA) authorized the use of CEM as an adjunct to FFDM and/or other ultrasound modalities with the purpose of localizing a known or suspected lesion [9,10]. In this study, a retrospective comparative analysis was completed to assess the diagnostic accuracy between CEM, FFDM, and histopathology. The sensitivity was 90.6% for FFDM and 98.12% for CEM. Additionally, CEM has superior specificity compared to FFDM, at 76.19% and 66.7%, respectively. The low NPV of FFDM, at 65.12%, was clearly inferior to the high NPV of CEM, at 91.43%. The improvement in diagnostic accuracy on the part of CEM lies in its ability to detect additional lesions and characterize them based on enhancement. The PPV of CEM and FFDM were similar, at 94.01% and 91.19%, respectively. The AUC of CEM was superior to that of FFDM, with a value of 0.87 (p < 0.001).

FFDM had a low sensitivity, especially in dense breast parenchyma, at 88.79% and a specificity of 70%, whereas CEM showed a higher sensitivity, specificity, and NPV, measuring 99.14%, 76.67%, and 95.83%, respectively. In comparison with FFDM alone, various incremental benefits of CEM were observed in 121 of the 150 cases. In the remaining 29 cases, CEM was concordant with FFDM with no additional benefit. The benefit of CEM was predominantly observed in the superior assessment of disease extent in 74 cases, additional lesion detection in 54 cases, confirmation of spurious asymmetric densities in FFDM as usual parenchyma in 100 cases, characterization of a lesion as benign/malignant in 46 cases, and estimation of residual viable disease in post-neoadjuvant chemotherapy in 13 cases.

As the study was conducted in a tertiary cancer care center, our results reflect the benefit of CEM in high-risk patients presenting with palpable lumps. Evaluation of additional satellite lesions that add to the extent of disease has become a priority in the oncology setting. Our study shows that CEM is superior to FFDM in evaluating disease extent, additional satellite lesion detection, and post-neoadjuvant chemotherapy viability assessment in patients with known breast cancer. The extent of microcalcifications seen in FFDM must be calculated in conjunction with the extent of disease seen in recombined subtracted images of CEM, as this calculation can indirectly aid in improved surgical planning for conservative surgeries. CEM has a high NPV, where the absence of enhancement strongly correlates to a benign pathology. This feature of CEM can be utilized to prevent unnecessary interventions in benign lesions and exclude malignancy in contralateral breasts.

CEM would be of great value in ruling out ambiguous findings in dense breast parenchyma. Prudent use of CEM in dense breast parenchyma aids in better estimation of disease extent and satellite lesion detection, thereby enhancing appropriate management for conservating surgeries. Mammographically and clinically occult lesions may be detected in contralateral dense breast parenchyma.

To date, there have been few studies on CEM published in international medical journals. Those that have been published had diversities in methodology, whereby most have been retrospective and few prospective. Additionally, the extant research has study population as a limitation, as most of the studies have been undertaken in smaller populations. In comparison with previous studies on CEM, our study had similar sensitivity and superior mean specificity. In one of the initial studies, performed by Dromain et al., the sensitivity and specificity were 93% and 58%, respectively [13]. In a study on 113 patients by Lobbes et al., CEM had a sensitivity of 100.0% and a specificity of 87.7% in comparison with mammography [14]. The diagnostic accuracy of CEM was superior to digital mammography in referrals from breast cancer screening. Additionally, this study by Lobbes et al. showed a high NPV for CEM, similar to our study, suggesting benignity in the absence of contrast enhancement [14].

CEM has shown greater sensitivity and specificity compared with other established modalities in several studies. For example, an increment of 4% in sensitivity (97%) and 34% in specificity (70%) as compared with FFDM was reported by Lalji et al., and an increment of 6% in specificity (95%) and a difference of 1% in sensitivity (94%) as compared with MRI was observed by Fallenberg et al. [15,16]. When comparing CEM with FFDM alone and with supplemental USG, few studies have shown greater sensitivity and specificity in identifying breast cancer [15]. Thus, it is in these areas that CEM appears promising. Most of the other studies have shown similar sensitivity for CEM, ranging between 90% and 100%. In our study, CEM showed superior sensitivity and specificity in comparison with FFDM. The ability to detect additional lesions increased sensitivity and the ability to characterize a lesion as benign or malignant increased the diagnostic accuracy of CEM. Table 10 shows the similarities between a small number of previous studies and the current study.

**Table 10:** Comparison of current study with previous studies

Study	Sample size	Sensitivity	Specificity
Current study	150	98.12%	76.19%
Dromain et al. [13]	120	93%	58%
Lobbes et al. [14]	113	100%	87.7%
Tardivel et al. [17]	195	84%	77%
Lalji et al. [15]	199	96.9%	69.7%
Richter et al. [18].	118	99%	54%

Mori et al. conducted a study comparing the diagnostic efficacy of CEM and FFDM with histopathology as the gold standard in dense breast parenchyma. In this study by Mori et al., CEM showed a sensitivity of 86.2% and specificity of 94.2%, with a diagnostic accuracy of 90.9%. On the other hand, digital mammography showed a sensitivity of 53.4%, a specificity of 85.9%, and a diagnostic accuracy of 72.7%. Our study showed a higher sensitivity but slightly lower specificity compared to the study by Mori et al. on dense breast parenchyma. CEM was shown to decrease the occurrence of false negative results, especially in dense breast parenchyma [19].

Overall, the analysis showed multifaceted superiority in the performance of CEM as compared with FFDM and USG - not only in detection but also in characterization and the management of breast cancer.

The strength of our study was a higher percentage (67%) of dense breasts and thus a higher prevalence of inconclusive or occult findings, limiting the diagnostic evaluation of FFDM and generating the need for additional imaging. In this context, this study has effectively analyzed the incremental value of CEM as compared with FFDM. As the study was conducted in a tertiary oncology care institute, the sample was representative of patients referred with a strong suspicion of breast cancer. This further establishes the supplemental role of CEM in detecting and characterizing additional lesions and the extent of disease in diagnostic mammography.

The fact that the study was conducted in a tertiary cancer care center is a limitation due to the referral bias, meaning a greater proportion of malignant cases. Similarly, rather than a retrospective study on patients with inconclusive FFDM, a prospective study on all patients in the diagnostic mammography category would eliminate selection bias.

The current recommendation for CEM is to use it as an additional problem-solving modality, especially in dense breasts. Studies in screening populations will be helpful in determining the true specificity of this modality. Larger and prospective studies are recommended to establish the use of CEM in all new cases with dense breast parenchyma.

## Conclusions

The diagnostic accuracy of CEM is greater than that of FFDM, as the superior sensitivity and outstanding high NPV of CEM as compared with FFDM make it a preferable supplemental modality, especially in dense breast parenchyma. Our findings conclude that CEM is superior to FFDM in the detection and characterization of breast cancer, detection of additional satellite lesions, and, hence, understanding of disease extent.

In low-middle-income countries, where FFDM is predominantly used in a diagnostic setting, despite supplemental USG, additional functional imaging is often required in inconclusive cases. Our study proposes CEM as a promising, readily available modality to empower anatomic FFDM imaging with functional imaging to improve its performance in the detection and management of breast cancer.
